# Rewired Neuroactive Ligand-Receptor Signaling Confers Adaptive Resistance to BCL-2 Inhibition in AML

**DOI:** 10.21203/rs.3.rs-8427312/v1

**Published:** 2026-01-12

**Authors:** Hiroaki Koyama, Sachiko Seo, William Tse, Sicheng Bian, Shujun Liu

**Affiliations:** Case Western Reserve University; Tokyo Women’s Medical University; Case Western Reserve University; Case Western Reserve University; Case Western Reserve University

## Abstract

A major challenge in treating AML with the BCL-2 inhibitor venetoclax is the frequent development of drug resistance, which diminishes therapeutic efficacy and leads to patient death. The fundamental mechanisms underlying this resistance are not fully understood. Here, we established venetoclax-resistant cell models of AML that propagate even when the levels of BCL-2, MCL-1, cleaved PARP, and cleaved caspase-9 are reduced, suggesting a BCL-2-independent resistance mechanism. Compared to sensitive cells, resistant Kasumi-1 (VENK) and MV4-11 (VENM) cells exhibit enhanced proliferation both *in vitro* and *in vivo*, forming larger and more numerous spheroids and colonies, and displaying higher tumorigenicity in mice. RNA sequencing and KEGG pathway analysis identified the neuroactive ligand–receptor interaction (NLRI) pathway as a key vulnerability in both resistant cell lines. While the NLRI pathway contains numerous altered genes, CHRNB4 is the only gene commonly shared and significantly downregulated in both VENK and VENM cells and tumors. Enforced expression of CHRNB4 in resistant cells with low basal expression impaired cell adhesion and colony formation. Clinically, CHRNB4 downregulation is associated with poor AML patient overall survival and predicts a diminished response to venetoclax treatment. This study identifies the NLRI pathway as a crucial vulnerability in venetoclax resistance and unveils CHRNB4 as a promising predictive biomarker for treatment response. These results suggest that targeting the NLRI pathway represents a novel strategy for developing next-generation therapies to improve the poor outcomes of current combination treatments.

## Introduction

Acute myeloid leukemia (AML) is a heterogeneous disease caused by diverse genetic and epigenetic changes in immature blood cells, leading to varied clinical presentations and prognoses. Despite advances, the outlook for AML patients, especially the elderly or those with high-risk features like FLT3-ITD or PTPN11 mutations, remains poor, as they are often unable to tolerate intensive chemotherapy.(1) The survival of AML cells heavily depends on anti-apoptotic proteins, most notably BCL-2. This dependency has made BCL-2 inhibitors, such as venetoclax (VEN), a paradigm-shifting and standard-of-care option for patients ineligible for intensive chemotherapy, achieving high remission rates and improved survival. Despite its efficacy, a significant portion of patients (~ 35%) develop primary resistance or eventually relapse.(2, 3) Mechanisms of resistance are multifaceted and include the co-expression of other anti-apoptotic proteins (e.g., MCL-1, BCL-XL) not targeted by the drug,(4, 5) the plasticity of the AML cell state leading to adaptive resistance,(6, 7) and the presence of other high-risk mutations (e.g., FLT3-ITD, PTPN11).(8–10) Combining VEN with other agents (hypomethylating agents (HMA) like azacitidine or decitabine, or low-dose cytarabine) has improved response rates in older, unfit AML patients.(3) However, overcoming primary resistance and preventing relapse still remain major clinical challenges due to the complex scenarios underlying HMA/VEN treatment.(11, 12)

The neuroactive ligand-receptor interaction (NLRI) pathway is a crucial signaling system in which neuroactive ligands bind to receptors to regulate neurobiological functions. Dysregulation of this pathway is associated with various neurodegenerative and psychiatric disorders. Beyond these established roles, the NLRI pathway is now recognized for its significant contribution to cancer, specifically enhancing tumor aggressiveness and drug resistance in solid malignancies like prostate and colon cancer.(13–16) NLRI’s oncogenic effects are driven by multiple mechanisms. It can promote cell growth by activating pro-survival pathways, including AKT and NFκB.(17) NLRIs also drive neuroendocrine differentiation and modulate the tumor microenvironment to foster immune suppression.(13, 18) Furthermore, a crosstalk between neuroactive pathways and epigenetic regulators, such as EZH2, can alter gene expression and foster a more aggressive, drug-resistant phenotype. Despite this understanding, significant knowledge gaps remain, including the specific, unidentified ligands and receptors involved in these oncogenic pathways, the precise molecular mechanisms that link neuroactive signaling to the development of drug resistance, and the complex interplay between NLRI pathways and other cancer-related signaling, such as hormonal and growth factor signaling. While the broader role of NLRIs in cancer is increasingly recognized, their specific contributions to aggressive leukemia and resistance to VEN in AML are not yet clear.

Considering the established role of the NLRI pathway in activating caspases, and the activation of caspases in VEN-induced cell death, we hypothesized that, upon exposure to VEN, the distinct levels of NLRI-associated cells will be reprogrammed. This reprogramming would potentially allow for the downregulation of key genes within this pathway, thereby inhibiting apoptosis and promoting an alternative, BCL-2-independent survival mechanism that confers resistance to VEN therapy. To test this hypothesis, we modeled and characterized VEN resistance across distinct *in vitro* and *in vivo* leukemia models. Utilizing comprehensive molecular, cellular, and animal studies, we demonstrate that VEN resistance is mechanistically linked to the downregulation of CHRNB4, a key gene we identified within the downregulation of CHRNB4, a key gene in NLRI pathway. Our findings indicate that CHRNB4 depletion predicts shorter survival time and reduced clinical responses to VEN treatment, whereas its overexpression inhibits the colony formation of these resistant cells. These results suggest that targeting the NLRI pathway represents a novel strategy for developing next-generation therapies to improve the poor outcomes currently associated with existing VEN treatments.

## Results

### VEN resistance is independent of the BCL-2 pathway

To dissect the molecular mechanisms of acquired VEN resistance, we established drug-resistant derivatives of the AML cell lines MV4-11 and Kasumi-1 via long-term culture with escalating concentrations of VEN (MV4-11: 0.001 to 0.01 nM; Kasumi-1: 0.001 to 0.08 nM). Cells cultured in parallel without drugs served as parental/sensitive controls (hereafter, PAR). We established resistance criteria as routine proliferation in medium containing 0.01 nM (MV4-11) and 0.08 nM (Kasumi-1) VEN, respectively, yielding the resistant cell lines MV4-11-VENR (VENM) and Kasumi-1-VENR (VENK).

**Figure 1:**
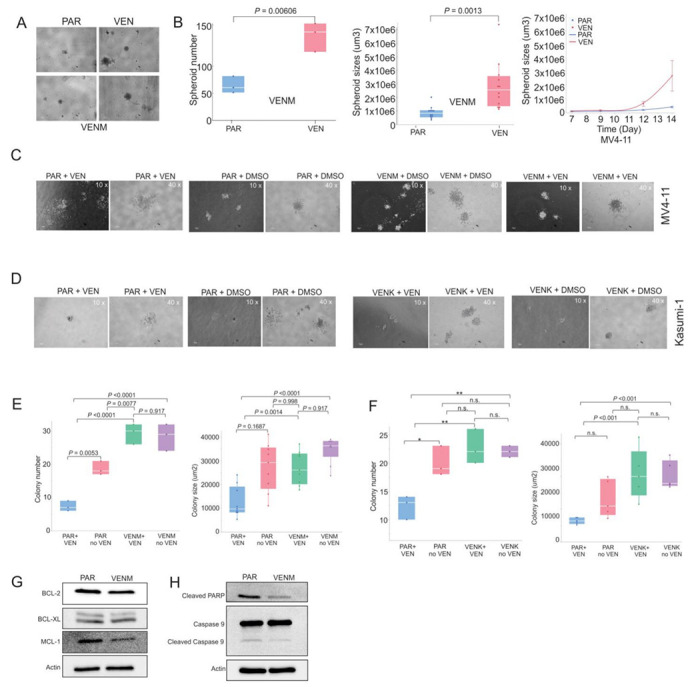
BCL-2 pathway is not required for the survival of VEN-resistant AML cells **(A, B)** Spheroid formation assays of VENM and PAR cells. (**A**) Representative images of spheroids captured on day 14. (**B**) Left: Quantification of spheroid number and size on day 14. Data shown are mean ±standard deviation. Right: Dynamic changes in spheroid size over the 14-day culture period. (**C, D**) Representative images of colonies captured on day 14 from colony formation assays comparing VENM, VENK, and respective PAR cells, with or without Venetoclax treatment. (**E, F**) Quantification of colony number and size on day 14 (mean ±standard deviation). (**G, H**) Western blot analysis showing expression changes of caspases and anti-apoptotic proteins in VENM cells compared to PAR cells. Results represent three independent experiments. Note: VEN, venetoclax; VENM, MV4-11 VEN resistant cells; VENK, Kasumi-1 VEN resistant cells; PAR, parental/sensitive cells; n.s, not statistically significant; *P<0.05; **P <0.01.

First, we measured the ability of the cells to form spheroids, three-dimensional structures that model tumor microenvironment (TME) interactions.(19) While PAR MV4-11 cells formed irregular, dendritic-like spheroids with less cohesive structure, the resistant cells developed a significantly greater number and larger size of spheroids compared to PAR cells (Fig. 1A, 1B). Furthermore, the resistant clones showed stronger cell-cell adhesion and maintained a more compact, spherical morphology, irrespective of VEN exposure. Notably, we failed to observe any spheroid formation in either the PAR or the VENK cells. Second, we assessed colony formation potential. In the presence of VEN, PAR cells exhibited poor colony formation and reduced adhesive capacity. In contrast, both the VENM and VENK formed more numerous, larger colonies with distinct morphologies (Fig. 1C, 1D). Resistant clones maintained this robust colony-forming potential even after VEN treatment, showing no significant changes in colony morphology or number, which confirms their resistance to VEN. Third, we confirmed the acquisition of resistance via drug sensitivity assays. While VEN treatment significantly reduced, or showed a trend toward reducing, the colony number and size of PAR cells, both the VENM and VENK cells showed minimal changes in colony number and size, indicating reduced sensitivity (Fig. 1E, 1F). These results suggest that selective pressure from VEN enhances the oncogenic potential and drug resistance of AML cells.

To investigate the mechanisms underlying these VEN-altered phenotypes, we performed Western blot analysis in VENM cells. We found that the expression levels of the anti-apoptotic proteins BCL-2 and MCL-1 are decreased compared to PAR cells; notably, BCL-xL levels remained unchanged (Fig. 1G). Furthermore, the levels of cleaved PARP and caspase-9 were also decreased, indicating an inhibition of the caspase pathway in the VEN-resistant cells (Fig. 1H). These results suggest that the acquisition of VEN resistance is accompanied by a shift toward alternative survival pathways rather than continued dependence on the canonical BCL-2 pathway.

### Resistant VENM cells exhibit enhanced tumor growth in vivo

**Figure 2:**
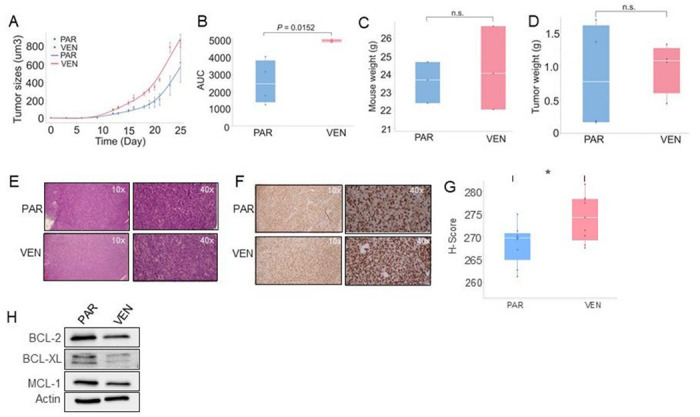
VEN-resistant AML cells exhibit increased tumorigenicity *in vivo* Approximately 1.0 × 10^6^ PAR and VENM cells were subcutaneously injected into the flanks of nude mice to assess tumor growth and properties (n = 4 tumors/group). (**A**) Tumor volume was measured over a 25-day period, showing dynamic changes in size. (**B**) Quantification of tumor growth represented as Area Under the Curve (AUC) values over the 25 days. (**C, D**) Changes in host body weight (**C**) and excised tumor weight (**D**) at the study endpoint (day 25). (**E, F**) Representative images of tumor sections analyzed by H&E staining (**E**) or IHC using an anti-Ki-67 antibody (**F**). All images were captured at ×200 magnification (n = 3). (**G**) Quantitative analysis of the Ki-67 IHC results presented in panel F. (**H**) Western blot analysis evaluating the protein expression levels of BCL-2, BCL-XL, and MCL-1 within the harvested tumor tissues. VEN, venetoclax; VENM, MV4-11 VEN resistant cells; H&E, Hematoxylin and eosin; IHC, immunohistochemistry staining; PAR, parental/sensitive cells.

To examine if VENM cells exhibit greater proliferative activity than their PAR counterparts *in vivo*, 3 × 10^6^ PAR or VENM cells were allowed to recover in drug-free medium for 72 hours before being subcutaneously injected into the flanks of nude mice. The tumor engraftment rate was similar in both groups (4/6 engrafted, 67%). Tumors derived from VENM cells exhibited markedly faster initial growth compared with those derived from PAR cells. While the proportional rate of increase in tumor volume was similar between both groups after the initial phase (before 8 days post injection), the early, faster growth of the VENM group resulted in a larger final tumor volume (over the 25-day experimental period) (Fig. 2A). This led to a significantly higher area under the curve (AUC) (Fig. 2B), which indicates a greater overall tumor burden or faster cumulative growth rate than that seen in tumors from PAR cells. No obvious changes in mouse body weight were observed (Fig. 2C). At the termination of the experiment, an obvious difference in tumor growth was observed between the treatment groups. The mean tumor volume was 860.1 ± 73.6 mm^3^ (mean ± SEM) in the VENM group compared to 615.4 ± 218.3 mm^3^ (mean ± SEM) in the PAR group (P = 0.35). This difference in growth rate was further supported by the significant larger mean AUC values: 4,932.9 ± 81.5 (mean ± SEM) in the VENM group vs 2,540.0 ± 1,276.0 (mean ± SEM) in the PAR group (P = 0.033). Furthermore, the tumor weight tended to be heavier in the VENM group (976 ± 188 mg) than those in the PAR group (840 ± 395 mg, P = 0.77) (Fig. 2C). This finding aligns with the higher *in vitro* proliferation observed in the VEN-resistant cells, which formed larger and more numerous spheroids and colonies. This enhanced proliferative capacity of the resistant-derived tumors was consistently confirmed by stronger staining for both H&E (Fig. 2E) and the proliferation marker Ki-67 (Fig. 2F, 2G) when compared with the PAR tumors.

To investigate the mechanisms underlying this enhanced tumorigenesis, we performed Western blotting. The results revealed that the expression of the anti-apoptotic markers BCL-2, MCL-1, and BCL-xl is slightly decreased in VENM vs control tumors (Fig. 2H), consistent with our *in vitro* findings. Taken together, these data indicate that VENM cells acquire more aggressive phenotypes *in vivo*, independent of BCL-2 pathway.

### The NLRI pathway is dysregulated in VENM and VENK cells

**Figure 4:**
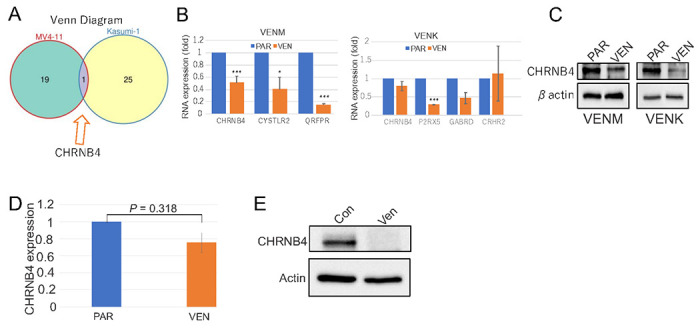
Downregulation of CHRNB4 expression in *in vitro* VENM cells and *in vivo*VENM-derived tumors (**A**) Venn diagram illustrating the overlap of DEGs between VENM and VENK cells. (**B, C**) qPCR (**B**) and Western blot (**C**) analysis demonstrating the relative expression levels of the indicated genes (specifically including CHRNB4) in the generated resistant cell lines. The Western blot results represent the findings from three independent experiments. (**D, E**) qPCR (**D**) and Western blot (**E**) for the expression of indicated genes in tumors. The results in Western blot represent three independent experiments. VEN, VEN resistant cells; VENM, VEN resistant MV4-11 cells; VENK, VEN resistant Kasumi-1 cells.

Among the enriched pathways identified by transcriptomic analysis, the NLRI pathway exhibited the most pronounced dysregulation in both VENM and VENK cells. This pathway involved a total of 20 genes in VENM cells and 26 genes in VENK cells (Supplementary Table 6), where CHRNB4, a key representative component of the NLRI pathway, is shared by both VENM and VENK cells (Fig. 4A). We used qPCR to further validate the expression changes of specific NLRI-related genes in VENM and VENK cells. Specifically, we analyzed CHRNB4, CYSLTR2, and QRFPR in VENM cells, and CHRNB4, P2RX5, GABRD, and CRHR2 in VENK cells. This analysis revealed that CHRNB4, CYSLTR2, and QRFPR are significantly downregulated in VENM cells compared to PAR cells. In contrast, only P2RX5 is significantly downregulated in VENK cells vs PAR cells, with CHRNB4 and GABRD showing a trend of downregulation and CRHR2 exhibiting no significant changes (Fig. 4B). To corroborate these transcriptomic findings at the protein level, we examined the expressions of the key gene CHRNB. We observed a marked reduction in CHRNB4 protein levels in both VENM and VENK cells compared to PAR cells (Fig. 4C). Importantly, the RNA and protein expression of CHRNB4 decreased in VENM tumors relative to PAR tumors (Fig. 4D, 4E). These results consistently suggest that the dysregulation of the NLRI pathway, specifically the suppression of CHRNB4, may play a role in the development of resistance to VEN.

### Downregulation of CHRNB4 in VENM and VENK cells is associated with VEN resistance

We investigated the role of the NLRI pathway in regulating resistance to VEN, focusing specifically on the gene CHRNB4. This focus was driven by key observations: First, CHRNB4 was the only gene commonly altered in both VENM and VENK cells within the NLR pathway (see Fig. 4A); Second, while the impacts of CHRNB4 on cancer pathogenesis and tumorigenesis in smoking-mediated cancers like head and neck squamous cell carcinoma (HNSCC) and esophageal squamous cell carcinoma (ESCC) has been reported,(20–22) its potential functional contribution to cancer drug resistance, particularly leukemia resistance, had not been previously established.

**Figure 5:**
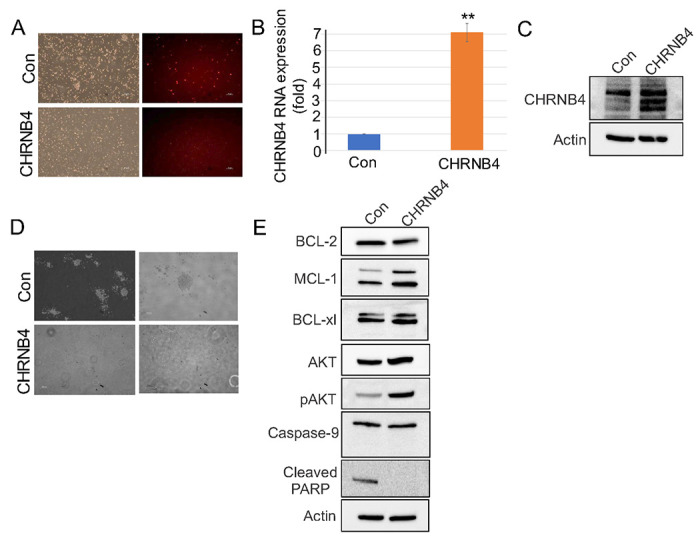
Impact of CHRNB4 overexpression on the characteristics of VENM cells (**A**) Successful CHRNB4 overexpression was confirmed in VENM cells following lentiviral infection with a CHRNB4 expression vector and subsequent selection using 2 μg/mL puromycin. Representative fluorescence microscopy images illustrate the robust expression of the linked reporter, indicative of successful transduction. (**B, C**) qPCR (**B**) and Western blot (**C**) for the expression of CHRNB4 in cells from (**A**). (**D**) Colony-formation assays were performed to assess the impact of sustained CHRNB4 expression on cell proliferation and clonogenic survival, comparing CHRNB4-overexpressing cells to control cells. (**E**) Western blot analysis of cell lysates was conducted to evaluate protein expression levels of CHRNB4 and relevant indicated target genes in overexpressing cells relative to controls. The immunoblots shown are representative of results obtained from three independent experiments.

To investigate the functional consequences of CHRNB4 modulation on VENM cell growth, we overexpressed CHRNB4 in VENM cells which exhibit lower levels of endogenous CHRNB4 expression (Fig. 5A). The successful increase in CHRNB4 expression was validated at both the RNA level via qPCR and the protein level via Western blot (Fig. 5B, 5C). We then performed colony-forming assays to determine the effects of CHRNB4 overexpression on resistant cell growth. We found that CHRNB4 overexpression markedly reduces both cell adhesion and colony formation capacity compared to control groups (Fig. 5D). Critically, this inhibitory effect was observed irrespective of further treatment VEN application; that is, the addition of treatment VEN to CHRNB4-overexpressing cells caused no additional significant changes in cell growth compared to untreated CHRNB4-overexpressing cells, suggesting that restored CHRNB4 function is the primary promoter of growth suppression in this context.

Previous studies showed that CHRNB4 mediates cancer cell proliferation and survival through an active PI3K/Akt/mTOR pathway or inhibited apoptosis.(20, 21) To further investigate the role of CHRNB4, we performed Western blot analysis in CHRNB4-overexpressing cells. This analysis revealed increased levels of pAKT and MCL1, while decreasing the levels of cleaved PARP, with no changes in cleaved caspase-9 (Fig. 5E). These results confirmed the activation of potent survival pathways and the continued suppression of apoptosis. Surprisingly, however, functional assays presented a notable paradox. Despite the robust activation of these pro-survival signals, we observed a marked reduction in both colony formation and cell adhesion. This finding reveals a significant dissociation between the elevated biochemical survival signaling and the impaired cellular function induced by CHRNB4 overexpression.

### CHRNB4 downregulation predicts shorter survival time and less VEN response

**Figure 6:**
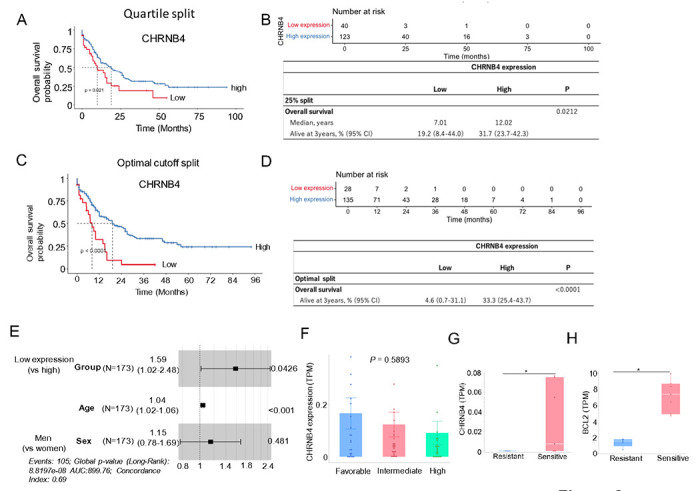
Prognostic significance of CHRNB4 expression in AML and its association with VEN responses (**A-D**) Analysis of AML patient data from the TCGA Firehose Legacy study (n=sample size). (**A**) Kaplan-Meier (KM) survival curve demonstrating significantly poorer overall survival in patients with low CHRNB4 expression (≤25th percentile). (**B**) Forest plot identifying low CHRNB4 expression as an independent adverse prognostic factor. (**C, D**) KM curves confirming consistent OS trends when data is stratified by median split (**C**) and optimal cut-off (**D**). (**E**) Hazard ratios and 95 percent confidence intervals for overall survival from patient group with low expression of CHRNB4 compared to those with high CHRNB4 expression. (**F**) Comparison of CHRNB4 expression across favorable, intermediate, and poor prognostic subgroups (defined by FLT3/NPM1 status). No statistically significant differences were observed, though expression trended lower in the poor-prognosis group. (**G, H**) Analysis of RNA-sequencing data from an independent cohort of 12 AML clinical samples (GSE132511). (**G**) Correlation between CHRNB4 and BCL-2 expression levels. (**H**) Association between CHRNB4 expression and clinical response to VEN treatment.

To assess the clinical significance of CHRNB4 in AML, we analyzed the TCGA Firehose Legacy AML cohort (n = 173). Patient characteristics are presented in Supplementary Table 7. Kaplan–Meier survival analysis revealed a significant association between low CHRNB4 expression levels and shorter overall survival (OS) (*P* = 0.021) (Fig. 6A–6E). Specifically, patients in the lowest quartile (25%) for CHRNB4 expression had a median OS of 7.01 months, compared with 12.02 months for patients with higher expression levels. Furthermore, a multivariate Cox proportional hazards regression confirmed that low CHRNB4 expression is an independent adverse prognostic factor in AML (Hazard Ratio [HR] = 1.59, 95% Confidence Interval [CI] = 1.0154–2.476, *P* = 0.0426). To verify the consistency of these findings, we performed additional survival analyses using both median and optimal cut-offs (determined via maximally selected rank statistics), all of which consistently demonstrated poorer survival in the low-expression group. These results are further supported by the observation that CHRNB4 expression is downregulated in high-risk AML patients (e.g., those with FLT3-ITD without NPM1 co-mutation) compared with favorable-risk patients (NPM1 without FLT3-ITD co-mutation) (Fig. 6F). Together, these findings suggest that the downregulation of CHRNB4 may play a critical role in mediating VEN resistance in AML.

The role of CHRNB4 in regulating sensitivity to VEN was investigated using RNA-seq data (GSE132511) from 12 AML clinical samples. Samples were categorized into VEN-sensitive (monocytic lineage, n = 5) and VEN-resistant (primitive lineage, n = 7) groups based on the established criteria.(11) Comparative analysis revealed that patients with higher BCL2 expression are more sensitive to VEN therapy compared to those in the lower-expression group (Fig. 6G). Furthermore, CHRNB4 expression was significantly lower in the VEN-resistant group compared to the sensitive group (P = 0.0332; Fig. 6H). This differential expression suggests that the downregulation of CHRNB4 may contribute to the mechanism of VEN resistance, highlighting its potential impact on drug efficacy *in vivo*.

## Discussion

VEN monotherapy yields modest outcomes in AML patients, facing multiple key challenges, including that most patients eventually relapse due to acquired resistance.(23, 24) Consequently, the FDA approved VEN only for combination use with HMAs or LDAC.(23, 25) These combinations improve response rates, remission, and overall survival, particularly in older patients.(26–28) However, challenges persist, including severe infections, myelosuppression, and ongoing treatment resistance.(26, 29) This study investigated the molecular mechanisms behind VEN resistance. Our data reveals that chronic VEN exposure rewires the NLRI pathway, downregulating the key gene CHRNB4. This effect mediates reduced VEN response and resistance development. These findings underscore the potential of NLRI pathway inactivation, specifically CHRNB4, as a predictive biomarker for VEN responses, and targeting this pathway as a therapeutic strategy for resistant AML.

The mechanisms of VEN resistance in leukemia are multifactorial, driven by molecular changes that restore the cell’s anti-apoptotic defenses. The primary mechanisms involve the upregulation of alternative survival proteins like MCL-1 and BCL-XL, or acquired mutations in BCL-2, BAX, and other signaling pathways like PI3K/AKT pathway.(30) In line with previous findings,(31, 32) we showed that both VENM and VENK are proliferate faster *in vitro*, as evidenced by the increased number and sizes of colonies and spheroids. Furthermore, xenograft mouse models revealed that VENM cells have higher tumorigenic potential *in vivo*. However, mechanistically, we found a slight decrease, but not an increase, in the expression of anti-apoptotic proteins MCL-1 and BCL-XL compared to PAR cells, which inherently lowers the cell’s threshold for apoptosis. Paradoxically, we observed a significant reduction of the cleaved caspase-9 and PARP, indicating an overall inhibition of the caspase pathway in the VEN-resistant cells. Collectively, these results suggest that acquiring VEN resistance involves a compensatory shift toward activating alternative survival pathways, moving away from dependence on the intrinsic BCL-2 pathway. Such discoveries provide novel sound rationales for combining VEN with other apoptosis-induced drugs, like HMAs, which further downregulate the expression of anti-apoptotic proteins including MCL-1 and BCL-xL, induce the expression of pro-apoptotic proteins like NOXA and reverse the methylation-mediated silencing of the GSDME gene.

FLT3 and KIT mutations are significant contributors to VEN resistance in AML.(33, 34) The presence of these high-risk mutations results in markedly diminished responses to VEN and a heightened potential for relapse following treatment.(8–10) Our studies specifically revealed that CHRNB4 expression is downregulated in high-risk AML patients (e.g., those with FLT3-ITD without NPM1 co-mutation) when compared to patients with a favorable risk profile (NPM1 without FLT3-ITD co-mutation). These findings support a potential collaborative effect between CHRNB4 depletion and FLT3 or KIT mutations in establishing VEN-resistant AML cell populations. This project focuses on the role of CHRNB4, a key gene that is downregulated in both Kasumi-1 (KIT mutation positive) and MV4-11 (FLT3 mutation positive) cell lines resistant to VEN. While CHRNB4 is the primary focus, each of these two resistant cell lines also exhibits altered expression in over 20 other genes within the NLRI inflammatory pathway. Future studies will further characterize these identified VEN resistance genes functionally and molecularly. This approach aims to identify novel molecular mechanisms and biomarkers of VEN resistance specifically within the context of FLT3 or KIT mutations.

The NLRI pathway regulates neurobiological functions, but its dysregulation also significantly contributes to tumor aggressiveness and drug resistance across various cancers.(13–16) General mechanisms, such as pro-survival pathway activation, neuroendocrine differentiation, and epigenetic crosstalk, are recognized; however, significant knowledge gaps persist regarding the precise molecular mechanisms linking NLRI to drug resistance, particularly the specific contributions to aggressive leukemia and resistance to VEN in AML. To address this knowledge gap, we performed RNA sequencing on VENM and VENK cells, as well as their respective PAR cells. KEGG and GSEA analyses identified the NLRI and apoptosis pathways centered at the top of both the down- and up-regulated transcripts in both VENM and VENK cell lines. The NLRI pathway encompasses 20 genes in VENM and 26 genes in VENK cells compared to the PAR cells. Intriguingly, the gene CHRNB4 is shared by both VENM and VENK cell lines and is consistently downregulated both *in vitro* and in tumors, suggesting that it could be a crucial regulator in the development of VEN resistance. Indeed, we observed that CHRNB4 is downregulated in VEN-resistant cells and tumors. These resistant cells exhibited characteristics of increased aggressiveness, including faster growth rates *in vitro*, larger and more numerous spheroid and colony formation, and higher tumorigenicity in mice models. Critically, when CHRNB4 was overexpressed in the VEN-resistant cells, both cell adhesion and colony formation were significantly inhibited. Importantly, lower expression of CHRNB4 in AML patients predicts shorter survival times and reduced response to VNE treatment. These findings identify a novel pathway, the NLRI pathway, as a key contributor to the development and maintenance of VEN resistance, and establish CHRNB4 as both a new predictive biomarker for VEN response and a promising therapeutic target for overriding VEN-resistant AML cells.

We found that low expression of the CHRNB4 gene correlates with a poor prognosis in TCGA data and is associated with molecular markers of BCL2 inhibitor resistance, such as elevated pAKT and reduced BCL2/MCL1. This suggests that the downregulation of CHRNB4 contributes to tumor progression and survival signaling. However, data from overexpression experiments reveal a seeming paradox: while molecular survival markers like pAKT and MCL1 increased upon CHRNB4 overexpression, functional outcomes were suppressed, evidenced by markedly reduced colony formation and adhesion, and a lack of cleaved PARP. This suggests that despite the upregulation of these specific survival signals, the overall functional survival capacity of the cells is impaired. A potential explanation for this “signal versus functional discrepancy” is that the excessive overexpression of CHRNB4 triggers severe cellular stress responses, such as ER stress and disrupted Ca^2+^ homeostasis.(35, 36) This chronic, high level of stress could induce an adaptive unfolded protein response, leading to the observed increases in pAKT and MCL1, which are survival signals, while simultaneously suppressing functional outcomes like proliferation and adhesion.(37–39) The reduction in cleaved PARP in this context would then reflect cells resisting apoptosis but being functionally compromised due to the overwhelming stress, which warrants further investigation.

## Experimental Methods

### Cell lines and cell culture

Leukemia cell lines, MV4-11 (CRL-9591) and Kasumi-1 (CRL-2724), were purchased from the American Type Culture Collection (ATCC). MV4-11 is a human acute myeloid leukemia (AML) cell line, derived from a male child with biphenotypic B-myelomonocytic leukemia. It is non-adherent and carries a t(4;11) translocation (MLL-AF4 fusion gene) and a FLT3-ITD mutation. Kasumi-1, derived from the peripheral blood of an Asian male patient with AML, is also an AML cell line. It exhibits characteristics of myeloid and macrophage lineages and carries a t(8;21) translocation (AML1/ETO fusion gene) and a c-KIT activating mutation. Cell lines were not authenticated or tested for mycoplasma in-house upon receipt but were verified against the International Cell Line Authentication Committee (ICLAC) database of commonly misidentified cell lines and were not found on the list.

Cells were cultured in RPMI-1640 medium (GE Healthcare #SH30027.01) supplemented with 10% (for MV4-11) or 20% (for Kasumi-1) fetal bovine serum (FBS, Gibco by Life Technologies #16140-071) and 1% Antibiotic-Antimycotic (Gibco by Life Technologies #15240062). Cells were maintained at 37°C in a humidified incubator with 5% CO_2_.

### Reagents and plasmid constructs

The BCL-2 inhibitor Venetoclax (HY-15531) was purchased from MedChemExpress. For *in vitro* studies, all drugs were initially dissolved in dimethyl sulfoxide (DMSO) and stored at −80°C. The CHRNB4 overexpression plasmid was obtained from Vector Builder, and the corresponding control plasmid was acquired from Addgene.

### Stepwise induction of venetoclax resistance

Parental MV4-11 and Kasumi-1 cells were exposed to a low, sub-lethal concentration of the BCL-2 inhibitor venetoclax (0.001 nM) in a stepwise manner to induce resistance. Surviving cells were cultured, and upon recovery of consistent growth rates, the venetoclax concentration was incrementally increased. Final concentrations reached 0.01 nM for MV4-11 and 0.08 nM for Kasumi-1 cells. The total selection period was 8–10 weeks. Cell viability, proliferation rate, and morphological changes were routinely monitored during the adaptation process. Cells were considered resistant when they could consistently proliferate in medium containing their respective final concentrations of venetoclax. Once established, resistant cell lines were routinely maintained in medium containing 0.01 nM (MV4-11) and 0.08 nM (Kasumi-1) venetoclax to preserve the resistant phenotype. For experiments requiring drug-free conditions, both parental and resistant cells were washed once with blank RPMI 1640 medium (no FBS, no antibiotics) to remove any residual venetoclax. The washed cells were then cultured in regular RPMI 1640 medium (with 10% FBS and antibiotics) for 48–72 hours to allow for recovery and proliferation in a drug-free environment before use in downstream assays.

### Lentivirus vector, virus production and virus infection

For lentivirus production, HEK-293T cells were seeded in a 10 cm culture dish at a density of 2.0 × 10^6^ cells cells/dish and incubated for 24 hours. Cells were subsequently transfected with 4 μg of either the target or scrambled control plasmids using a CalPhos^™^ Mammalian Transfection Kit, following the manufacturer’s protocol. Viral supernatants were collected at both 48 and 72 hours post-transfection and concentrated using a Lenti-X^™^ Concentrator (Clontech, Cat. #631232). For infection, 2 × 10^6^ cells recipient cells were resuspended in 1 ml of medium and transduced with the harvested lentiviruses in the presence of Polybrene at a final concentration of 4 μg/ml. Stable transformants were selected by adding puromycin to a final concentration of 1 μg/ml 24 hours post-transduction.

### Colony-forming assays

For colony-forming assays, cells were first suspended in 0.3 ml of IMDM medium (Stem Cell Technologies #36150) before being mixed with 3 ml of MethoCult^®^ medium (Stem Cell Technologies #03434). The cell-medium mixture was then dispensed into 35 mm dishes, and colony counts and sizes were recorded after 7–14 days.

### 3D Spheroid-forming assays

For spheroid assays, 2000 cells were suspended in 100 μL of Corning Matrigel Matrix Growth Factor Reduced (Corning #356230) and seeded on the 24-well culture plate. The spheroids were then cultured for 3 to 14 days under standard conditions. Imaging was performed using a Zeiss LSM 880 confocal/multiphoton microscope. Two-photon excitation was achieved with an 800 nm laser, and fluorophores were spectrally separated by their emission for detection.

### Western blotting

The whole cellular lysates were prepared by harvesting the cells in 1 × cell lysis buffer [20 mM HEPES (pH 7.0), 150 mM NaCl and 0.1% NP40] supplemented with 1 mM phenylmethane sulfonyl fluoride (PMSF, Sigma #10837091001), 1 × Phosphatase Inhibitor Cocktail 2 and 3 (Sigma #P5726, P0044), and 1 × protease inhibitors (protease inhibitor cocktail set III, Calbiochem-Novabiochem #539134). The proteins were resolved by sodium dodecyl sulfate (SDS)–polyacrylamide gel electrophoresis, transferred onto PVDF membranes (GE Healthcare #10600023), blocked by 5% non-fat milk followed by probing with first antibodies, anti-BCL-2 (Millipore, #05-729), anti-BCL-XL (Santa Cruz, #sc-7195), anti-MCL-1 (Santa Cruz, #sc-819), anti-AKT (Cell Signaling, #9272), Anti-pAKT (Cell Signaling, #4060), anti-CHRNB4 (Abcam, #ab233735), anti-β-Actin (Cell Signaling, #4967) as well as HRP-conjugated secondary antibodies, goat anti-rabbit IgG (Cell Signaling, #7074), goat anti-mouse IgG (Cell Signaling, #7076).

### RNA isolation, cDNA preparation and quantitative PCR (qPCR)

Total RNA was isolated according to the manufacturer’s instructions using the miRNeasy Kit (Qiaqen, #217004). Complementary DNA (cDNA) synthesis was synthesized from the isolated RNA using SuperScript^®^ III First-Strand Synthesis System (Invitrogen, #18080-051). Gene expression levels were quantified via qPCR using the SYBR Green Master Mix (Applied Biosystems, #4309155). Target gene expression was normalized to 18S ribosomal RNA levels. Primer sequences are CHRNB4: F 5’-CAGCTTATCAGCGTGAATGAGC-3’, R 5’-GTCAGGCGGTAATCAGTCCAT-3’; CYSLTR2: F 5’-ACTGAGGACCGTCCACTTGA-3’, R 5’-CCCAGCAAAGTAATAGAGCAGAG-3’; QRFPR: F 5’-CAGGCGCTTAACATTACCCC-3’, R CCGGTACAGAGCGATGAACTG-3’; P2RX5: F 5’-TACCTGGTCGTATGGGTGTTC-3’, R 5’-GCCCAAGATCCGAGGTGTTG-3’; GABRD: F 5’-GCATCCGAATCACCTCCACTG-3’, R 5’-GATGAGTAACCGTAGCTCTCCA-3’; CRHR2: F 5’-CCCTTGTCGTCAACTACCTGG-3’, R 5’-ACATTTCGCAGGATAAAGGTGG-3’; 18S: F 5’-ATTAAGGGTGTGGGCCGAAG-3’, R 5’-TGGCTAGGACGTGGCTGTAT-3’.

### RNA sequencing and data processing

Strand-specific transcriptome library construction was completed by enriching mRNA from total RNA, and sequenced by the DNBSEQ high-throughput platform at BGI (https://www.bgi.com). Briefly, mRNA was purified from total RNA using oligo(dT) magnetic beads, fragmented, and converted to double-stranded cDNA with dUTP incorporated into the second strand. The cDNA underwent end repair, A-tailing, and adapter ligation. UDG digestion was performed to remove the second strand before PCR amplification. After purification with XP Beads, the library was validated on an Agilent 2100 bioanalyzer. The double-stranded PCR product was heat-denatured and circularized using a splint oligo. Single-strand circular DNA libraries were amplified via rolling circle replication with phi29 polymerase to produce DNA nanoballs. These were sequenced on a patterned nanoarray using combinatorial probe-anchor synthesis, generating single-end 50 (or paired-end 100/150) base reads. Triplicate samples were sequenced.

Genes were considered significantly up- or down-regulated if they met a univariate p-value of < 0.05 and had an absolute fold difference of ≥ 1.5. A univariate significance threshold of 0.001 was used for filtering or pre-screening genes prior to comparison. Signaling pathway analysis was performed using DAVID (Version 6.7).

Significant KEGG pathways (adjusted P-value < 0.05) were visualized as dot plots using the clusterProfiler and ggplot2 R packages. The top up- or down-regulated categories were identified based on their statistical significance, and the number of genes contributing to each category was illustrated.

### In vivo tumorigenesis assays

Female athymic nude mice (4–6 weeks old) were purchased from the Jackson Laboratory. All animal procedures were conducted in accordance with the National Institutes of Health (NIH) guidelines for the care and use of laboratory animals and were approved by the Institutional Animal Care and Use Committee (IACUC) at Case Western Reserve University. Mice were monitored daily for signs of pain and distress, including significant weight loss, decreased activity, and hunched posture. All animals were provided ad libitum access to food and water throughout the study.

Parental or resistant cell lines (3 × 10^6^) were suspended in a 3:7 mixture of serum-free medium and Matrigel^®^ (Corning, Inc.) and subcutaneously injected into the flanks of 6- eek-old female athymic nude mice. Tumor growth was monitored every 2–3 days using an electronic caliper. Tumor volume was calculated using formula 1/2 × A × B ×B, where A is the length and B is the width, expressed in cubic millimeters. Tumor growth curves were generated for each treatment group by plotting the mean tumor volume over time and AUC was calculated. The study endpoint was determined per IACUC guidelines.

Upon reaching the study endpoint, mice were euthanized by CO_2_ inhalation followed by cervical dislocation. Excised tumors were divided for different analyses. A portion was snap-frozen and stored at −80°C for subsequent molecular characterization. Another portion was fixed in 10% neutral buffered formalin for 24 hours at 4°C for histological analysis. After fixation, tissues were transferred to PBS, processed, and embedded in paraffin blocks for immunohistochemical and hematoxylin & eosin staining.

### Hematoxylin and eosin (H&E) and immunohistochemical (IHC) staining

Tumor and tissue samples were harvested from mice and immediately preserved by fixation in 10% neutral buffered formalin. Following fixation, the samples were submitted to the Department of Pathology at the MetroHealth System, Case Western Reserve University, for processing and IHC (Ki-67) or H&E staining. The prepared slides were visualized and photographed with a Leica microscope fitted with a high-resolution spot camera, which was interfaced with Image-Pro Plus software for image acquisition.

### Gene expression and survival analysis

mRNA expression data for the CHRNB4 gene were obtained from The Cancer Genome Atlas (TCGA) AML cohort (TCGA, Firehose Legacy) via the cBioPortal for Cancer Genomics (https://www.cbioportal.org/). Patients were stratified into two groups based on their CHRNB4 mRNA expression levels: a low-expression group (lower 25% of expression values, i.e., the first quartile) and a high-expression group (remaining 75%). Overall survival (OS) was the primary endpoint. Survival curves were estimated using the Kaplan–Meier method, and differences between the groups were assessed using the log-rank test.

Survival analyses were conducted using the median expression value, optimal cutoff values, and a predefined 25% cutoff to stratify patients into expression groups. A multivariate Cox proportional hazards regression analysis was then performed to evaluate the prognostic impact of CHRNB4 expression alongside other relevant clinical variables. All statistical analyses were performed using JMP^®^ Student Edition version 18 (SAS Institute Inc., Cary, NC, USA).

### Statistical analysis

For statistical analyses, group means were compared using a two-tailed Student’s t-test. A non-parametric test was adopted for cases with unequal variance. A p-value of < 0.05 was considered statistically significant. All analyses were conducted using JMP Student Edition Version 18 software.

*In vitro* experiments, including qPCR, Western blotting, and cell proliferation assays, were performed in biological triplicate (n = 3) unless otherwise stated in the figure legends or main text. Sample sizes were not statistically predetermined and thus were not chosen to ensure adequate power to detect a pre-specified effect size. No blinding or randomization was used in the experimental procedures. For each figure, the specific statistical test used is justified as appropriate.

## Figures and Tables

**Figure 3:**
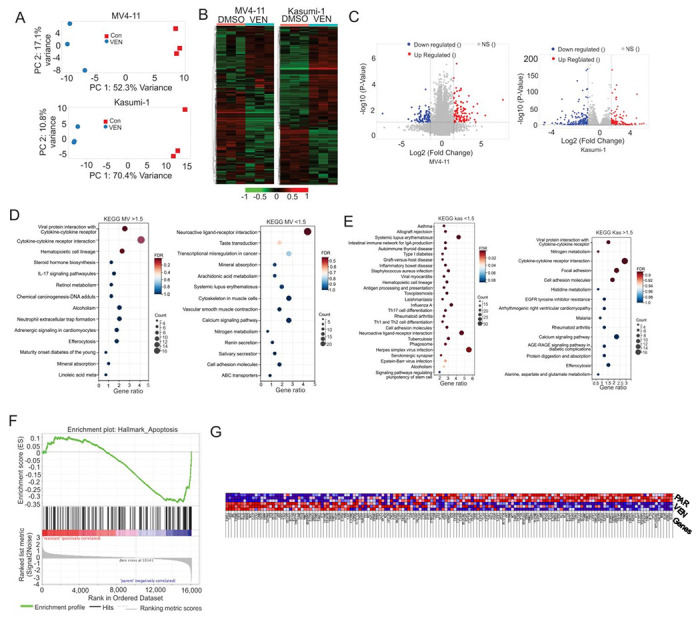
Selective pressures imposed by VEN significantly alter the transcriptomic landscapes in AML cells (**A**) PCA analysis illustrating the global gene expression profiles of all analyzed samples. The plot visualizes the overall variance and clustering of samples based on their transcriptomic signatures, demonstrating the distinct separation between PAR and VENM groups along PC1 and PC2. (**B**) Heatmaps displaying the patterns of DETs between treatment groups. (**C**) Volcano visualizing DETs based on statistical significance and magnitude of change. The plot highlights transcripts that are significantly upregulated (e.g., red dots) or downregulated (e.g., blue dots) in VENM cells compared to PAR. The x-axis represents the log_2_ fold change (log_2_FC), and the y-axis represents the negative log_10_ adjusted p-value (−log_10_ padj), with dashed lines indicating the significance thresholds applied (e.g., padj < 0.05 and |log_2_FC| > 1). (**D, E**) KEGG pathway analysis identifying biological pathways significantly enriched among the DETs. Bubbles display the top enriched pathways, ranked by significance (p-value). This analysis pinpoints the specific cellular processes and molecular functions most affected by VEN selective pressures, such as apoptosis regulation, NLRI pathway. (**F**) DSEA plot showing significantly enriched hallmark pathways and their leading-edge gene subsets that contribute most significantly to the enrichment of altered biological processes in resistant cell populations. (**G**) Heat map depicting relative gene expression levels within the leading-edge subset genes, with expression values ranging from red (high) to dark blue (lowest). PCA, Principal Component Analysis; DETs, differentially expressed transcripts; GSEA, Gene Set Enrichment Analysis; FC, fold change; FDR, False Discovery Rate.

## Data Availability

All relevant data supporting the key findings of this study were available from the corresponding authors upon reasonable request. Partial data were obtained from “Public data analysis” including TCGA.
